# Relating gene expression evolution with CpG content changes

**DOI:** 10.1186/1471-2164-15-693

**Published:** 2014-08-20

**Authors:** Huan Yang, Dawei Li, Chao Cheng

**Affiliations:** Department of Gynecology, The Ninth People’s Hospital of Chongqing, Chongqing, 400700 China; Department of Microbiology and Molecular Genetics, College of Medicine, Burlington, VT 05405 USA; HB7400, Remsen 702, Department of Genetics, Geisel School of Medicine at Dartmouth, Hanover, NH 03755 USA; Institute for Quantitative Biomedical Sciences, Norris Cotton Cancer Center, Geisel School of Medicine at Dartmouth, Lebanon, NH 03766 USA

## Abstract

**Background:**

Previous studies have shown that CpG dinucleotides are enriched in a subset of promoters and the CpG content of promoters is positively correlated with gene expression levels. But the relationship between divergence of CpG content and gene expression evolution has not been investigated. Here we calculate the normalized CpG (nCpG) content in DNA regions around transcription start site (TSS) and transcription terminal site (TTS) of genes in nine organisms, and relate them with expression levels measured by RNA-seq.

**Results:**

The nCpG content of TSS shows a bimodal distribution in all organisms except platypus, whereas the nCpG content of TTS only has a single peak. When the nCpG contents are compared between different organisms, we observe a different evolution pattern between TSS and TTS: compared with TTS, TSS exhibits a faster divergence rate between closely related species but are more conserved between distant species. More importantly, we demonstrate the link between gene expression evolution and nCpG content changes: up-/down- regulation of genes in an organism is accompanied by the nCpG content increase/decrease in their TSS and TTS proximal regions.

**Conclusions:**

Our results suggest that gene expression changes between different organisms are correlated with the alterations in normalized CpG contents of promoters. Our analyses provide evidences for the impact of nCpG content on gene expression evolution.

**Electronic supplementary material:**

The online version of this article (doi:10.1186/1471-2164-15-693) contains supplementary material, which is available to authorized users.

## Background

In vertebrates, CpG dinucleotides are substantially depleted compared to what would be expected by chance [1]. This is caused by the relatively high mutation rate from CpG to TpG. Deamination of cytosine gives rise to uracil, which, as a “foreign” nucleotide, is easy to be recognized and corrected by DNA repair system. However, when the cytosine in CpG sites is methylated, deamination of methylcytosine produces thymine, which cannot be recognized as foreign and thus less likely to be repaired [2]. As a consequence, hypermethylated DNA regions are more likely to lose CpG dinucleotides. In vertebrates, DNA methylation serves as an important mechanism for regulating gene expression, and a large fraction of CpG sites are methylated [3, 4], leading to an overall depletion of CpG dinucleotides in the genome [5]. In some DNA regions, however, the CpG sites are not methylated in germline cells and therefore are preserved or even over-represented [6–8]. These regions are termed as CpG islands (CGIs), which typically occur at or near the transcription start site of genes, particularly, in the vicinity of housekeeping genes [8]. In addition to DNA methylation, other evolutionary processes, such as biased gene conversion [9–11], have also been proposed to explain the evolution of GC% as well as the generation and maintenance of CGIs.

Paradoxically, there is still no satisfying definition for CGI. To identify them in a genome, arbitrary thresholds have been used [12]. For example, a widely applied definition of CGI is a region with ≥200 bp, GC% > 50%, and an observed-to-expected CpG ratio > 60% [12]. Based on the presence of CGI in the vicinity of promoters, genes can be divided into CGI-associated and non-associated. But again, there is no satisfying way to associate CGIs with genes. To address this issue in the context of promoter studies, Saxonov et al. defined a metric called normalized CpG (nCpG) content-- the ratio of the observed number of CpG dinucleotide to the expected number within a 3 kb region around the TSS of genes [13]. They found that human promoters displayed a bimodal distribution in their nCpG content, and therefore could be divided into two classes: high CpG promoters (HCPs) and low CpG promoters (LCP).

The relationship between GC% of genes and gene expression levels has been studied, which showed only a weak correlation [8, 14–16]. The normalized CpG content, however, has been reported to be highly predictive to the activities of promoters measured by systematic luciferase assays [17]. Normalized CpG content alone predicted the activities of ‘ubiquitously’ expressed promoters with high accuracy (R = 0.75, R is the correlation coefficient between predicted and actual activities). In our previous studies, we also found a high correlation between nCpG content of promoters and expression level of TSSs quantified by Cap Analysis of Gene Expression (CAGE) in human cell lines [18].

To understand phenotypic evolution, gene expression changes in different species have been studied based on microarray data [19–22] and more recently based on RNA-seq data [23]. It has been suggested that the divergence of gene expression is largely driven by the evolution of transcription factor binding sites [24–26]. Giving the high correlation between expression level and normalized CpG content of genes, we hypothesize that the expression divergence of genes should be reflected by the changes of CpG content in their promoters.

To test this hypothesis, we utilize the RNA-seq expression data in nine organisms and correlate the expression changes with nCpG content difference between different organisms. Our results suggest a positive correlation between them when two distantly related organisms are compared, e.g. human versus mouse. TSSs show a bimodal distribution in their nCpG contents diving them into high CpG and low CpG promoters, while there is only a single peak in the distribution of TTS nCpG content. We also observe different evolution patterns between TSS and TTS in their nCpG contents: TSSs exhibit faster divergence rates than TTSs in the nCpG content between closely related species, but are more conserved when distantly related species are compared. Our analysis provides new insights into the impact of nCpG content on gene expression evolution.

## Results

### Normalized CpG content of promoters in nine species

**Figure 1:**
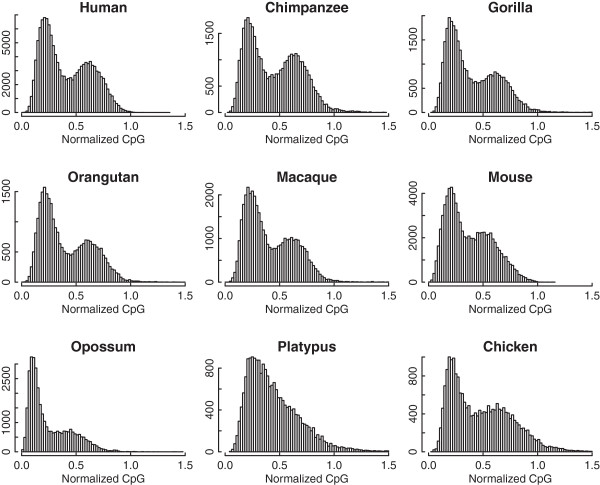
**Distribution of the normalized CpG content of promoters in nine organisms.** Note that a bimodal distribution is shown in all organisms except for platypus.

We investigate the nCpG contents of all promoters (3 kb centering on TSS) in 9 vertebrate species (human, chimpanzee, gorilla, orangutan, macaque, mouse, opossum, platypus and chicken). As shown in Figure 1, with the exception of platypus, we observe a bimodal distribution of the TSS nCpG contents, indicating the existence of two promoter classes. As a control, we also calculate the nCpG contents for all TTSs in the nine organisms. In contrast to TSS, the TTS nCpG contents (3 kb centering on TTS) in all organisms show a single-peak distribution, in which the high CpG peak observed in the TSS distribution is absent (Additional file 1). Absence of the high-CpG peak suggests that CpG sites around TTS are not protected from mutation by demethylation. In platypus, the absence of bimodality for TSS nCpG content is consistent with the observation of small CGI number in this organism reported by Pask et al. [27], and presumably caused by its extremely high GC%: 45.5% in platypus versus ~41% in eutherian and chicken genomes [28]. We also find that the TSS nCpG content varies considerably in different species. For example, human TSSs tend to have much higher nCpG content (mean = 0.41, median = 0.36) than opossum TSSs (mean = 0.25, median = 0.17), consistent with the fact that opossum genome possess low GC% and extremely low CpG dinucleotide density [29].

Since TSSs show a bimodal distribution in their nCpG content, we can divide promoters into two categories: the high CpG promoters (HCPs) and the low CpG promoters (LCPs). The cut-off value for such a categorization and the number of HCPs and LCPs in different species are summarized in Table 1. As shown, in most organisms the numbers of HCPs and LCPs are fairly comparable except for gorilla (21,260 LCPs versus 13,476 HCPs) and opossum (25,253 LCPs versus 9,456 HCPs).

**Figure 2:**
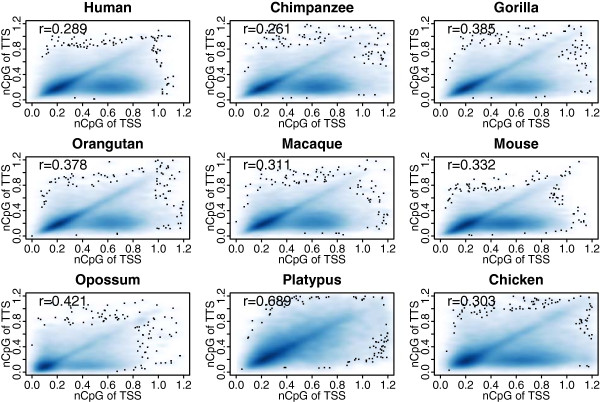
**Correlation of normalized CpG content between TSS and TTS in nine organisms.** Note that the correlation is much higher in platypus than in other organisms.

Next we examine the correlation of nCpG contents between TSS and TTS across all transcripts. We find weak correlations in eight of the nine organisms, ranging from 0.26 to 0.42 (Figure 2). Since the correlations in all organisms are calculated based on a large number of transcripts, all of them are highly significant. Strikingly, the correlation in platypus (r = 0.69) is much higher than in all the other species. Recalling the absence of HCP peak in its TSS nCpG content distribution (Figure 1), we posit that platypus has a different evolutionary scenario from other organisms in CpG usage: the CpG content appears to be less associated with by DNA methylation in this organism.

**Table 1:** **The normalized CpG contents of high CpG and low CpG promoters in nine organisms**

	#Transcript	Threshold	#LCP	#HCP	LCP		HCP	
					Mean	SD	Mean	SD
Human	134229	0.425	74761	59468	0.120	0.083	0.631	0.242
Chimpanzee	38878	0.414	19074	19804	0.152	0.081	0.654	0.241
Gorilla	34736	0.451	21260	13476	0.166	0.090	0.660	0.249
Orangutan	28057	0.441	16757	11300	0.136	0.086	0.650	0.246
Macaque	41617	0.444	24848	16769	0.141	0.085	0.646	0.259
Mouse	82775	0.411	49293	33482	0.120	0.089	0.588	0.230
Opossum	34709	0.361	25253	9456	0.123	0.082	0.524	0.151
Platypus	20972	na	na	na	na	na	na	na
Chicken	21561	0.437	10840	10721	0.231	0.087	0.720	0.253

#indicates the number of “transcripts”, “LCPs” or “HCPs”.

**Figure 3:**
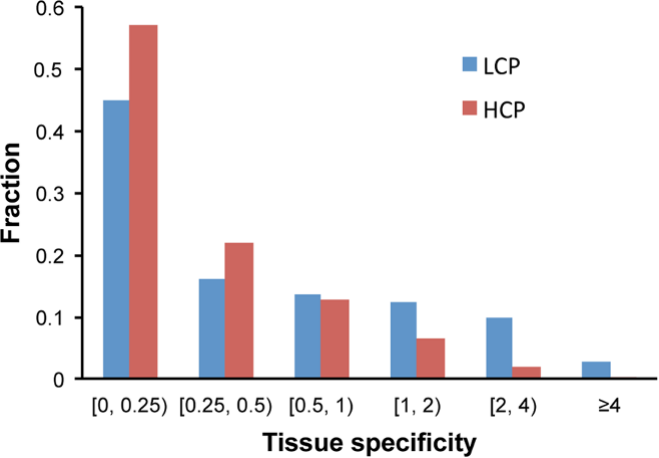
**Distribution of tissue specificity scores of mouse HCP and LCP genes.**

Previous studies have shown that HCP genes are more likely to be housekeeping genes while LCP genes tend to be tissue specific. We define a metric called tissue specificity score (TSPS) to quantify the relationship between tissue specificity and TSS nCpG content of genes. We calculate the TSPSs for all human and mouse genes. Overall, the TSPSs of genes show a weak negative correlation with their TSS nCpG content (e.g. r = −0.168 in mouse), verifying that genes with lower nCpG contents are more tissue specific (Additional file 2). The TSPSs of HCP genes are significantly lower than those of LCP genes (P = 7e-76, Wilcoxon rank sum test), with an average value of 0.35 and 0.72 in mouse, respectively. The distributions of TSPSs for HCP and LCP genes in mouse are shown in Figure 3. As shown, 45% LCP and 57% HCP genes have a TSPS < 0.25 (housekeeping); in contrast, 13% LCP and only <2% HCP genes have a TSPS > 2 (tissue specific). Similar results have also been observed in human. Our quantitative analysis confirms the relationship between promoter nCpG content and gene tissue specificity.

### Conservation of normalized CpG content

**Figure 4:**
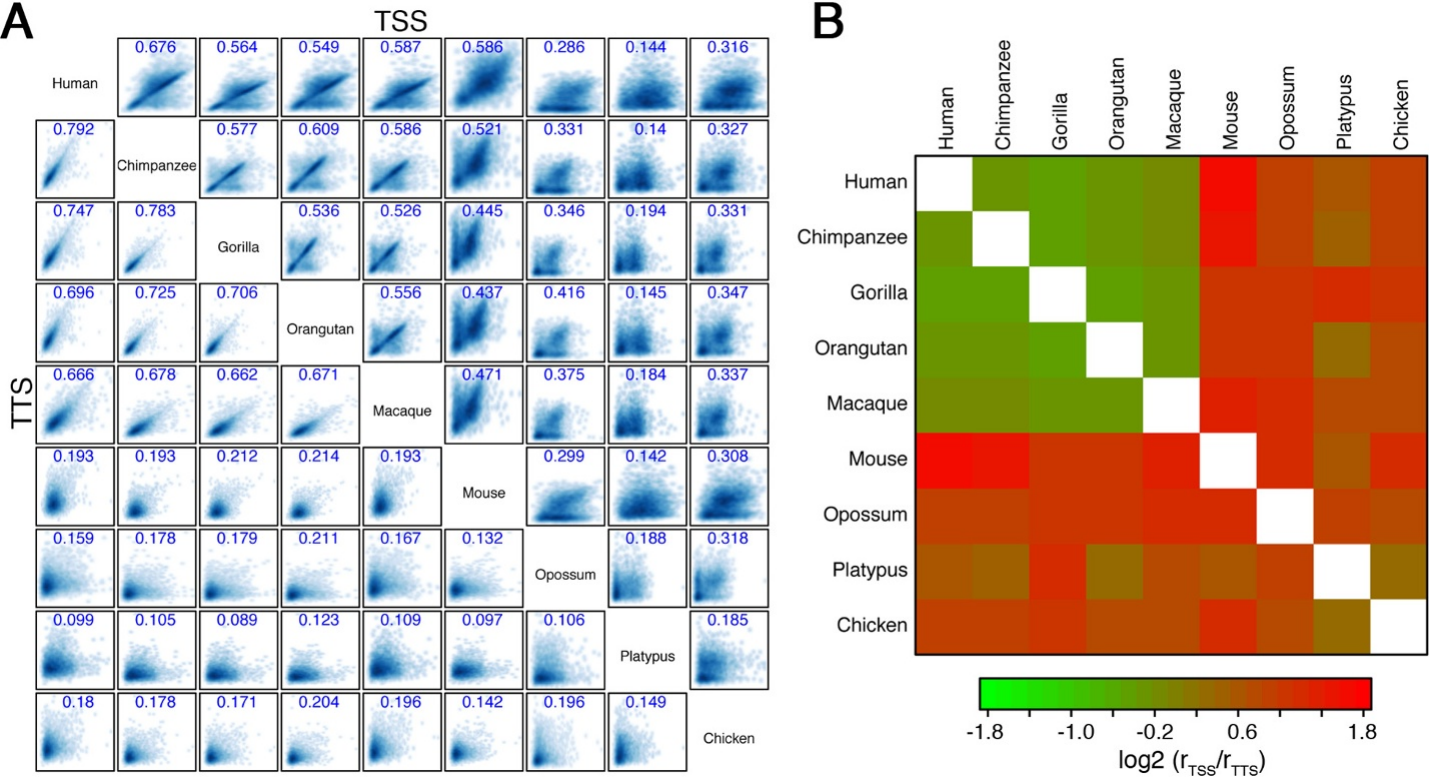
**Conservation of nCPG contents of TSS and TTS across different organisms.** **(A)** Pairwise correlation of nCpG contents for TSS (upper right) and TTS (lower left). The values are Spearman correlation coefficients. **(B)** nCpG content divergence of TSS relative to that of TTS. For each pair of organisms, the log2 ratio of r_tss_ (correlation of TSS nCpG content) to r_tts_ (correlation of TTS nCpG content) is shown.

To explore how the nCpG content of TSS and TTS diverged during evolution, we calculate their correlation coefficients between each pair of the nine organisms (Figure 4A). Interestingly, we observe higher correlations for TTS between closely related species, but higher correlations for TSS between distantly related species. As shown, the correlations of TSS are always lower than those of TTS between the five primate species (human, chimpanzee, gorilla, orangutan and macaque), indicating a faster divergence rate of TSS nCpG relative to TTS. However, when two distantly related organisms are compared, the nCpG content of TSS is more conserved than that of TTS. For example, the correlation coefficient of TSS between human and mouse is 0.586, much higher than the correlation coefficient of TTS (r = 0.193). This conservation pattern is more obvious when we use a heatmap to show the ratios of TSS correlations to TTS correlations for all pairs of organisms (Figure 4B). In the figure, one can observe a faster divergence rate of TSS nCpG relative to TTS nCpG within the primate group (i.e. a smaller log2(r_tss_/r_tts_)); and outside the group a much slower divergence rate of TSS nCpG. This reveals two facets regarding evolution of nCpG content of TSS: it may account for the divergence of gene expression in closely related species, while in distant species it is more conserved relative to TTS, presumably due to the possession of an enriched number of functional cis-regulatory elements [30].

In addition, we examine the conservation of HCP/LCP gene category between organisms. Specifically, for each pair of the eight organisms (excluding platypus) we select the orthologous gene pairs with only a single TSS in both organisms, and count the number of pairs that are HCP in both (HH), LCP in both (LL), and HCP in one but LCP in the other (HL and LH). Our results indicate that the HCP/LCP category is very conserved during the evolution (Additional file 3). As an example, for human versus mouse there are 277 HH pairs and 132 LL pairs, but only 54 HL pairs and 18 LH pairs. Namely, the majority of genes (85%) have a conserved HCP/LCP category between human and mouse (P = 7e-50, χ^2^ test).

### Correlation between normalized CpG contents and gene expression levels

It has been reported previously that nCpG content is correlated with expression level of genes [13, 18]. The availability of gene expression data in nine organisms enables us to make a more systematic investigation on this issue. We compare the expression levels of HCP and LCP genes in all tissues of the eight organisms (platypus is excluded) and confirm that HCP genes have significantly higher expression levels than LCP genes (Additional file 4). Compared to the HCP class, the LCP class has a larger fraction of non-expressed genes (expression is not detected by RNA-seq). Even after the non-expressed genes are excluded from comparison, HCP genes still show significantly higher expression levels than LCP genes.

We further explore the relationship between nCpG content and gene expression levels by directly computing their correlations. We calculate the Spearman correlation coefficients of gene expression levels with nCpG content of both TSS and TTS. As shown in Table 2, nCpG content is positively correlated with gene expression levels. This is the case for both TSS and TTS, but TSS is substantially more correlated than TTS, suggesting that they might be more functional in regulating gene expression.

**Table 2:** **Correlation of gene expression levels with nCpG contents of TSSs and TTSs**

	Brain		Cerebellum		Heart		Kidney		Liver		Testis	
	TSS	TTS	TSS	TTS	TSS	TTS	TSS	TTS	TSS	TTS	TSS	TTS
Human	0.574	0.317	0.560	0.324	0.528	0.297	0.537	0.300	0.504	0.292	0.563	0.610
Chimpanzee	0.556	0.228	0.557	0.194	0.524	0.135	0.520	0.168	0.499	0.155	0.517	0.109
Gorilla	0.503	0.191	0.504	0.185	0.473	0.165	0.464	0.134	0.441	0.131	0.484	0.118
Orangutan	0.500	0.197	0.477	0.165	0.456	0.131	0.467	0.166	0.439	0.133	na	na
Macaque	0.512	0.186	0.496	0.178	0.477	0.168	0.466	0.149	0.450	0.180	0.477	0.147
Mouse	0.671	0.421	0.669	0.419	0.609	0.383	0.601	0.363	0.560	0.343	0.571	0.352
Opossum	0.364	0.228	0.377	0.246	0.355	0.217	0.335	0.189	0.293	0.144	0.305	0.175
Platypus	0.308	0.306	0.309	0.307	0.306	0.292	0.299	0.276	0.272	0.256	0.328	0.281
Chicken	0.325	0.121	0.318	0.111	0.319	0.115	0.323	0.100	0.306	0.119	0.342	0.114

The values shown in the table are Spearman correlation coefficients.

We next extend our correlation analysis to human and mouse microarray data. Again, we observe positive correlations between CpG content of TSS and gene expression levels in all of the 79 human tissues and the 61 mouse tissues. But compared to the RNA-seq data, the correlations in microarray data are much lower, with the largest correlation coefficient r = 0.287 in human (Additional file 5) and r = 0.346 in mouse (Additional file 6). This might reflect the quality difference between RNA-seq and microarray expression data: RNA-seq data is known to be more sensitive and more accurate than microarray data [31, 32].

### Relationship between normalized CpG difference and gene expression evolution

**Figure 5:**
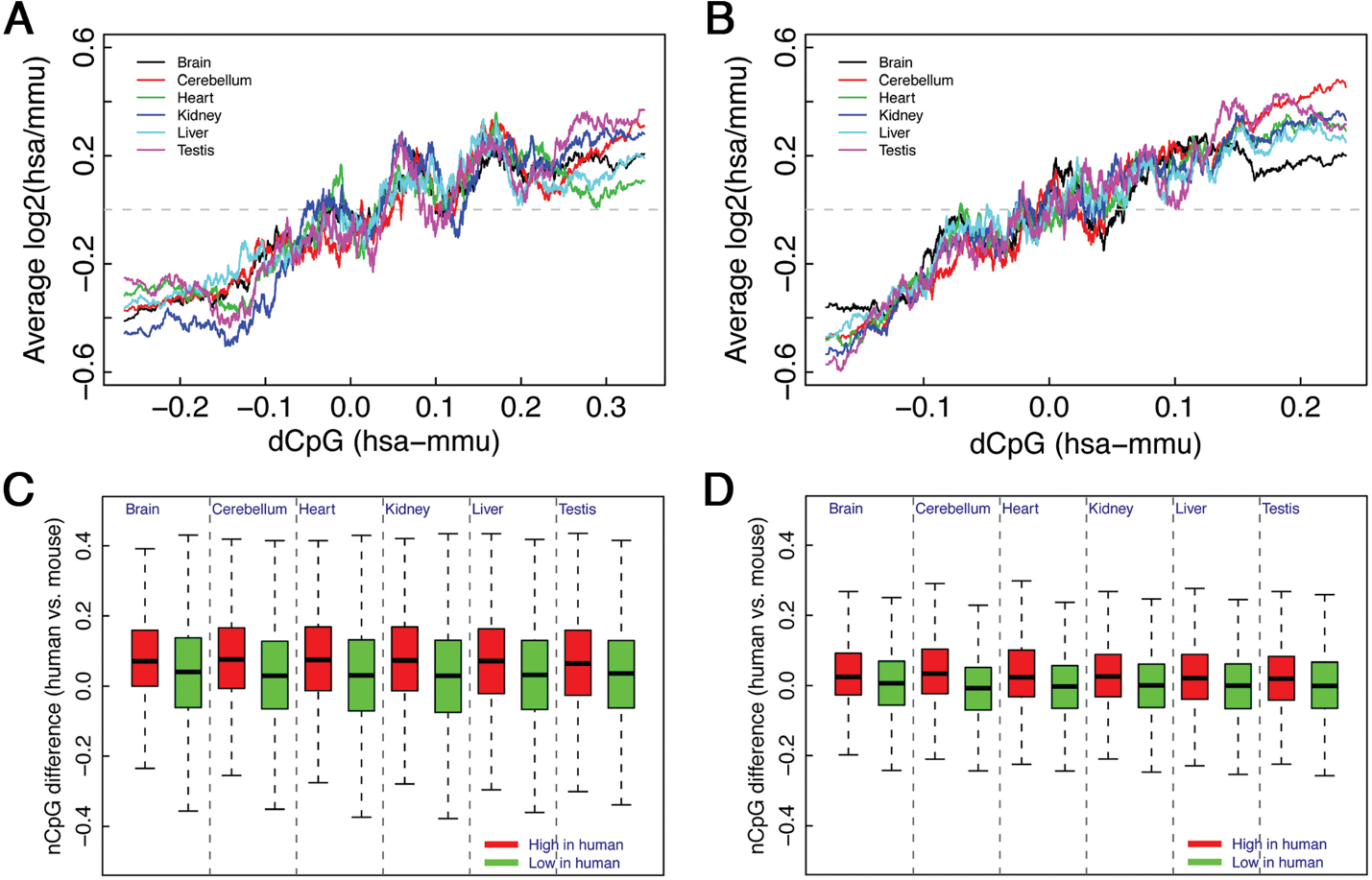
**Relationship between gene expression change and nCpG difference between human and mouse.** The increase of average expression change, log2(hsa/mmu), is accompanied with the increase of nCpG difference (dCpG) of TSS **(A)** and TTS **(B)**. Consistently, genes highly expressed in human (>two-fold change) have significantly larger nCpG difference (human versus mouse) in their TSS **(C)** and TTS **(D)** proximal DNA regions than those lowly expressed genes.

Having confirmed the correlation between CpG contents and gene expression levels, we then ask: can the evolution of gene expression be reflected by the divergence of CpG content between different organisms? To address this question, we calculate the TSS nCpG content difference (dCpG) between human and mouse othologous genes, and sort them in the increasing order. Then in each sliding window with 400 gene pairs, we calculate the average expression change in human versus mouse, log2(hsa/mmu). As shown in Figure 5A, we observe an obvious trend between dCpG of TSS and average expression change in all the six tissues. Interestingly, the trend is also observed for TTS (Figure 5B). These results suggest that the evolution of gene expression is accompanied with the CpG content change of genes in their TSS and TTS proximal DNA regions.

We perform the same analysis for all pair of organisms and confirm the relationship between CpG content change and gene expression divergence (Additional file 7). Such a relationship can be observed for TSS and TTS in all distantly related organism pairs. However, when two organisms are closely related (e.g. within the primate group), the trend is hardly detected, presumably, due to short divergence time.

When we identify the differentially expressed genes between human and mouse using two-fold as the threshold, we find that genes highly expressed in human have significantly larger nCpG content difference (human versus mouse) for both TSS and TTS (Figure 5C and 5D), which again confirms the relationship between CpG divergence and gene expression change. Note that due to a global increase of nCpG content of TSS in human relative to mouse, even genes lowly expressed in human tend to have higher nCpG content in their TSS proximal regions (dCpG > 0).

A similar trend analysis shown in Figure 5A is also performed by comparing human and mouse microarray expression data in matched tissues. However, when microarray data are used, we cannot detect the relationship between nCpG content difference and gene expression change described above (Additional file 8). The up-regulated group and the down-regulated group in human versus mouse identified based on microarray data do not show significant difference in their normalized CpG contents.

## Discussion

To study the impact of CpG islands (CGIs) on gene expression, most previous studies associated genes with nearby CpG islands to divide genes into two categories: CGI associated and non-associated. It is often tricky and arbitrary to determine the cut-off values for identifying CGIs and for associating them with genes. Here, we choose a different strategy by focusing on the TSS and TTS proximal DNA regions of genes. Generally, regulatory elements are highly enriched in TSS but not in TTS regions [33]. Here we include TTS as a control for TSS, since the TTS and TSS often share similar sequence features-- as shown by the high correlation in nCpG content between TSS and TTS in platypus. In eight of the nine organisms we observe a bimodal distribution of TSS nCpG content, suggesting that there are two different promoter classes: HCP and LCP. HCPs are enriched for CpG dinucleotide and in most cases are associated with a nearby CGI. In contrast to the bimodal distribution of TSS, there is only a single peak in the distribution of TTS nCpG content. In addition, We observe quite different evolution patterns between TSS and TTS in their nCpG content (Figure 3): between closely related species TSS diverged in a higher rate than TTS, while in distantly related species TSS are more conserved. These results reveal a dual character of promoters during evolution: they exert more impact on gene divergence, and meanwhile, they are subject to more selective constraints. This idea may be extended to CGIs, since they are the major contributors to high CpG content of HCPs. In line with this, CGIs have been shown to harbor many regulatory elements and are active regulators for transcription [34].

In the nine organisms, platypus exhibits a very different evolutionary pattern. First, the CpG content of platypus TSS does not show a bimodal distribution: the HCP peak is missing. Second, the correlation of nCpG contents between TSS and TTS in platypus is 0.689, much higher than all the other organisms. Third, in platypus TSS and TTS CpG contents have comparable correlations with gene expression levels; while in other organism TSS show a much higher correlation than TTS. Together with the fact that platypus has an extremely higher G + C% content (45.5%) and a smaller number of CGIs [28], this may suggest that the regulatory function and mechanism of DNA methylation in platypus is different from other species.

Our analysis shows a clear relationship between gene expression change and nCpG content divergence in two distantly related species, such as human versus mouse. Compared to down-regulated genes, genes up-regulated in human tend to have higher nCpG content relative to mouse in both TSS and TTS proximal DNA regions. Such a relationship is observed when RNA-seq is used to measure gene expression levels. However, the same analysis using microarray data fails to show such a relationship. Moreover, the correlation between microarray expression level of genes and nCpG content of promoters is very weak. The expression changes of orthologous genes in different species are often subtle and are complicated by many confounding factors issues such as cross-species normalization. For this reason, the relationship between gene expression change and nCpG divergence can only be revealed by RNA-seq data, which is more sensitive and precise than microarray data. On the other hand, the nCpG divergence between two species requires a long period of time for accumulating mutations. Thus the relationship can only be observed between distantly related species.

If the occurrence of CGIs and HCPs is merely a consequence of low DNA methylation rate of these DNA regions in germline cells, one may expect the correlation between nCpG content and gene expression levels to be observed only in germline cells. However, our study shows that such a correlation can be observed in all of the six tissues. This is because (1) expression profiles in different tissues are highly correlated and thus gene expression in non-germline tissues is overall similar to expression in germline cells; (2) more importantly, CGIs and HCPs are enriched for functional elements, which directly affect the expression level of genes. For example, the CpG binding protein CFP1 regulates histone modification through binding to DNA containing unmethylated CpG motifs and consequently affects gene expression [35]. CGIs are associated with specific DNA sequence features that are critical for their roles in regulating gene expression. On one hand, DNA sequence features associated with CGIs facilitate the formation of a transcriptionally permissive chromatin state in CGI associated promoters by destabilizing nucleosomes and attracting proteins [36]. In fact, most housekeeping genes are associated with CGIs in their promoters and these CGIs are generally unmethylated, whereas tissue specific promoters usually are not associated with CGIs. On the other hand, CGI associated promoters can be silenced through dense CpG methylation [37] or polycomb recruitment [29, 38], again using their distinctive DNA sequence composition.

It has been suggested that DNA methylation in promoter regions represses gene expression [39]. We calculated the correlation coefficients between gene expression and promoter methylation (from TSS to 200 bp upstream) across all transcribed genes in hESC and IMR90 cells using ENCODE data. We observed weak correlations with r = −0.37 in hESC and r = −0.22 in IMR90, which are much lower than the correlation coefficient between normalized CpG contents for TSS and gene expression levels in human. Many highly methylated genes are transcribed with high expression levels. Consistent with our observations, Du et al. reported a weak negative correlation between gene expression and promoter methylation in H1 cell line with r = −0.24 [40]. In addition, more recent studies have demonstrated that the across individual methylation-gene expression associations can be either positive or negative, even for DNA methylation sites in promoter regions [41, 42]. Despite the correlation between gene expression and DNA methylation, it remains unclear whether DNA methylation is the cause or the consequence of altered gene expression. In fact, recent studies showed that DNA methylation might be a passive reflection of transcription factor binding or a consequence of gene repression [43, 44]. This is supported by the negative correlation between transcription factor expression and the methylation levels of their binding sites [44], and by the depletion of cytosines within transcription factor binding sites [43]. In this study, we demonstrate a correlation between gene expression change and nCpG content divergence between distant species. It would be interesting to investigate whether and how DNA methylation is involved in such a relationship.

## Conclusion

In conclusion, comparative analysis in nine vertebrate organisms suggests that gene expression changes between organisms are correlated with the alterations in the normalized CpG contents of promoters. It provides evidences that support the impact of nCpG content change on gene expression evolution.

## Methods

### Gene expression data and DNA sequences

RNA-seq gene expression data were downloaded from Brawand *et al.*, which measured transcript and gene expression levels in six tissues (brain, cerebrum, heart, liver, kidney and testis) of nine organisms: human, chimpanzee, gorilla, orangutan, macaque, mouse, opossum, platypus and chicken [23]. Gene expression levels were represented as RPKM (reads per kilobase per million mapped reads) and were normalized so that levels of orthologous genes in different organisms are directly comparable [23]. For most tissues, expression levels in multiple samples were available in each organism. In these cases, we calculated their average at the log scale (log2 RPKM) to obtain the final expression levels. Microarray gene expression data for human and mouse were available from Su et al. [45], which contained expression levels of genes in 79 human tissues and 61 mouse tissues.

DNA sequences around TSS and TTS (−1.5 kb ~ 1.5 kb) of genes were extract from whole genome sequences. The genomic locations of transcripts in the nine organisms were determined based gene annotation from Ensembl database [46]. The Ensembl 57 assembly was used. The orthologous genes pairs are determined by referring to Brawand et al. [23].

### Calculation of normalized CpG content

For each transcript, normalized CpG contents (nCpG) of TSS and TTS were calculated based on DNA sequences of 3 kb (1.5 kb upstream to 1.5 kb downstream of a TSS/TTS). Normalized CpG content was defined as the ratio of observed number of CpG dinucleotide (observed CpG) to the expected number (expected CpG), and was calculated using the method described in Saxonov et al. [13]. Expected CpG was calculated as (GC content/2)^2^. Some genes possess multiple transcripts, which may have different TSS and/or TTS. In these cases, we used the average nCpG of these TSS/TTSs to represent the nCpG of the genes. Alternatively, the maximum nCpG contents of these TSS/TTSs were used to represent the nCpG content of the genes. These two definitions of TSS/TTS nCpG contents for genes resulted in consistent results and conclusions.

With the exception of platypus, the TSS nCpG contents in all organisms demonstrate a bimodal distribution. To define high CpG promoters (HCPs) and low CpG promoters (LCPs), we set the threshold in an organism as the nCpG contents at the lowest density between the two peaks in the distribution, with promoters on the right side as HCPs and promoters on the left side as LCPs.

### Calculation of tissue specificity score for genes

The tissue specificity of human and mouse genes was calculated based on their expression patterns in different tissues from the microarray data by Su et al. [45]. Given the expression pattern of a gene, we calculated a tissue specificity score (TSPS) to quantify the degree of tissue specific expression [47], which is defined as following:


where f_i_ is the ratio of the gene expression level in tissue i to its sum total expression level across all tissues, and p_i_ = 1/n for all tissues (n = 79 for human and n = 61 for mouse, which is the total number of tissues), is the fractional expression of a gene under a null model assuming uniform expression across all tissues. A larger TSPS value suggests more specific expression of a gene in a single or a few tissues, whereas a TSPS value of zero suggests uniform expression of the gene.

### Calculation of correlation coefficients

The Spearman correlation coefficient between TSS/TTS nCpG content and gene expression levels are calculated based on all genes in each organism. Similarly, the correlation coefficient r is calculated between TSS nCPG content and TTS nCpG content for each organism. The cross-organism spearman correlation coefficient of TSS or TTS nCpG content was calculated based on all orthologous gene pairs between two organisms.

The significance for a given correlation coefficient r is estimated based on the Fisher z-transformation. Specifically, we calculated , in which N is the total number of samples (e.g. the total number of genes for calculating correlation coefficient between TSS nCpG content and gene expression level in an organism). The p-value was then calculated by referring z to a standard normal distribution.

## Electronic supplementary material

Additional file 1: **Distribution of the normalized CpG content of TTS in nine organisms.** (PDF 32 KB)

Additional file 2: **Relationship between tissue specificity and normalized CpG content of TSS in mouse. HCP and LCP are genes with high CpG content and low CPG content promoters.** (PDF 932 KB)

Additional file 3: **Comparison of gene classes between species. H: high CpG promoter; L: Low CpG promoter.** (PDF 13 KB)

Additional file 4: **Expression levels of HCP and LCP genes in different tissues of different organisms.** (TIFF 948 KB)

Additional file 5: **Correlation of normalized CpG content with microarray gene expression levels in 79 human tissues.** (PDF 3 MB)

Additional file 6: **Correlation of normalized CpG content with microarray gene expression levels in 61 mouse tissues.** (XLS 36 KB)

Additional file 7: **Relationship between RNA-seq gene expression change and nCpG difference for all pair of organisms.** (XLS 30 KB)

Additional file 8: **Relationship between microarray gene expression change and nCpG difference for all pair of organisms.** (XLS 28 KB)
