# Supplementary figures and images for: Relating gene expression evolution with CpG content changes
Source: BMC Genomics. 2014 Aug 20;15(1):693. doi: 10.1186/1471-2164-15-693 (PMC4148958; doi:10.1186/1471-2164-15-693)

**Human**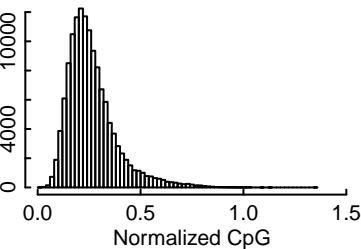**Chimpanzee**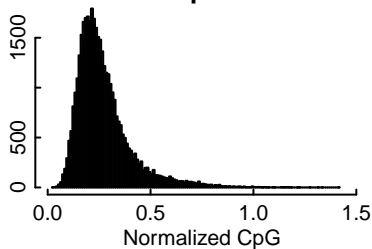**Gorilla**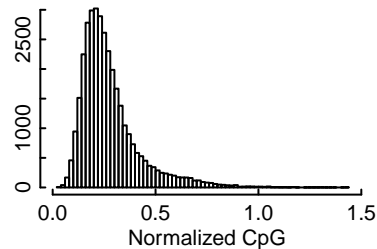**Orangutan**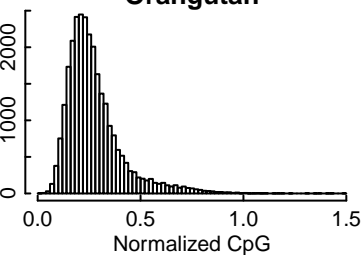**Macaque**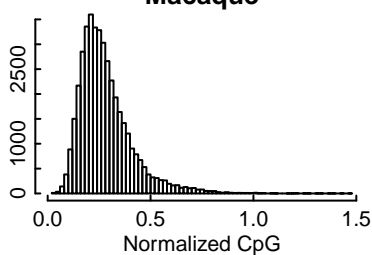**Mouse**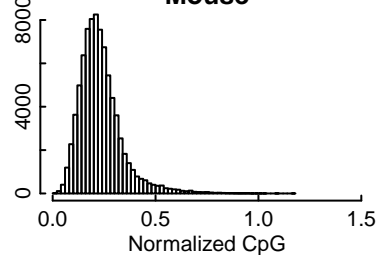**Opossum**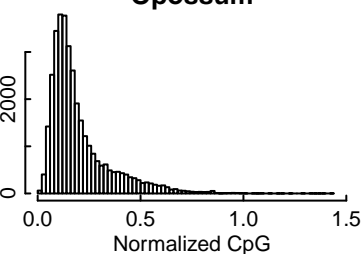**Platypus**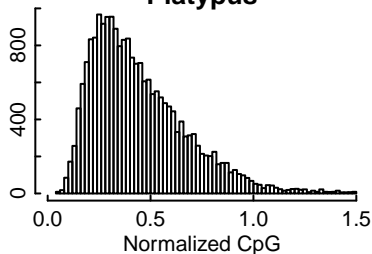**Chicken**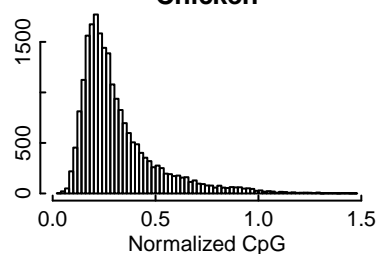

Supplement: Supplementary file 1 — Additional file 1: Distribution of the normalized CpG content of TTS in nine organisms. (PDF 32 KB) [file 12864_2013_6381_MOESM1_ESM.pdf]

**A**

Tissue specificity (log10)

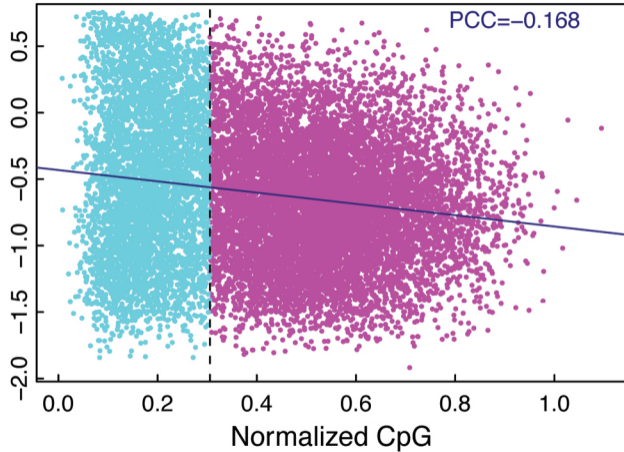**B**

Tissue specificity

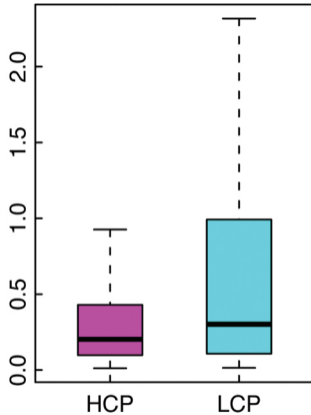

Supplement: Supplementary file 2 — Additional file 2: Relationship between tissue specificity and normalized CpG content of TSS in mouse. HCP and LCP are genes with high CpG content and low CPG content promoters. (PDF 932 KB) [file 12864_2013_6381_MOESM2_ESM.pdf]

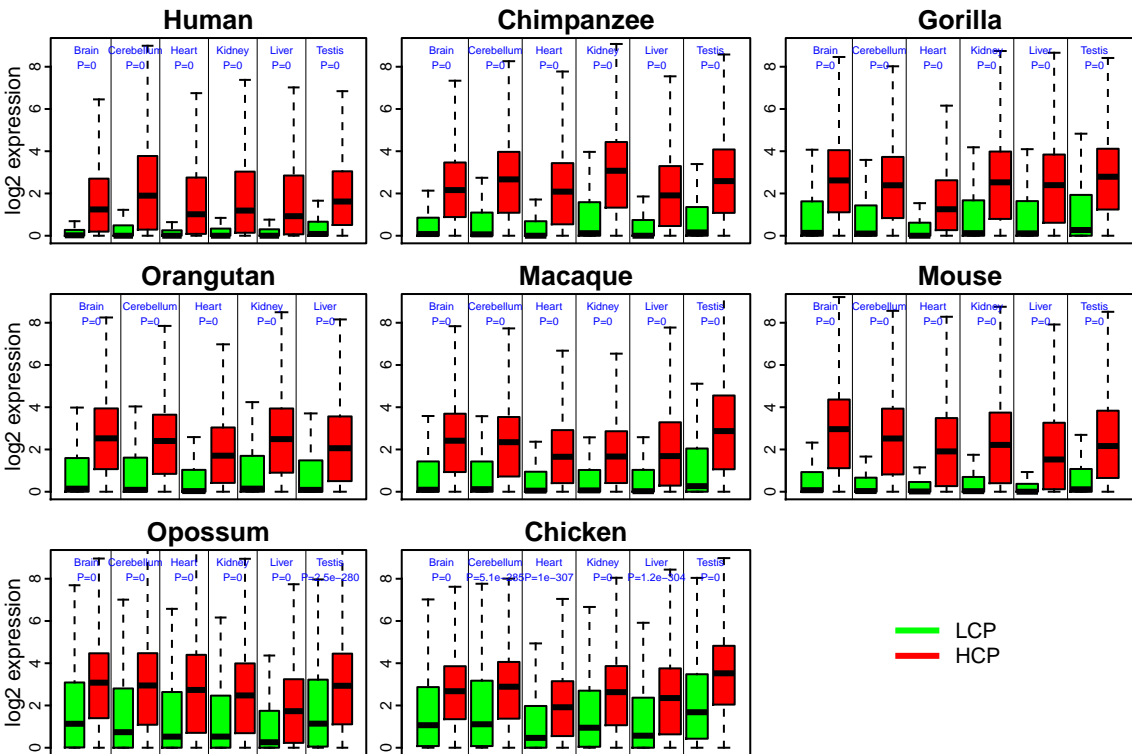

Supplement: Supplementary file 3 — Additional file 3: Comparison of gene classes between species. H: high CpG promoter; L: Low CpG promoter. (PDF 13 KB) [file 12864_2013_6381_MOESM3_ESM.pdf]

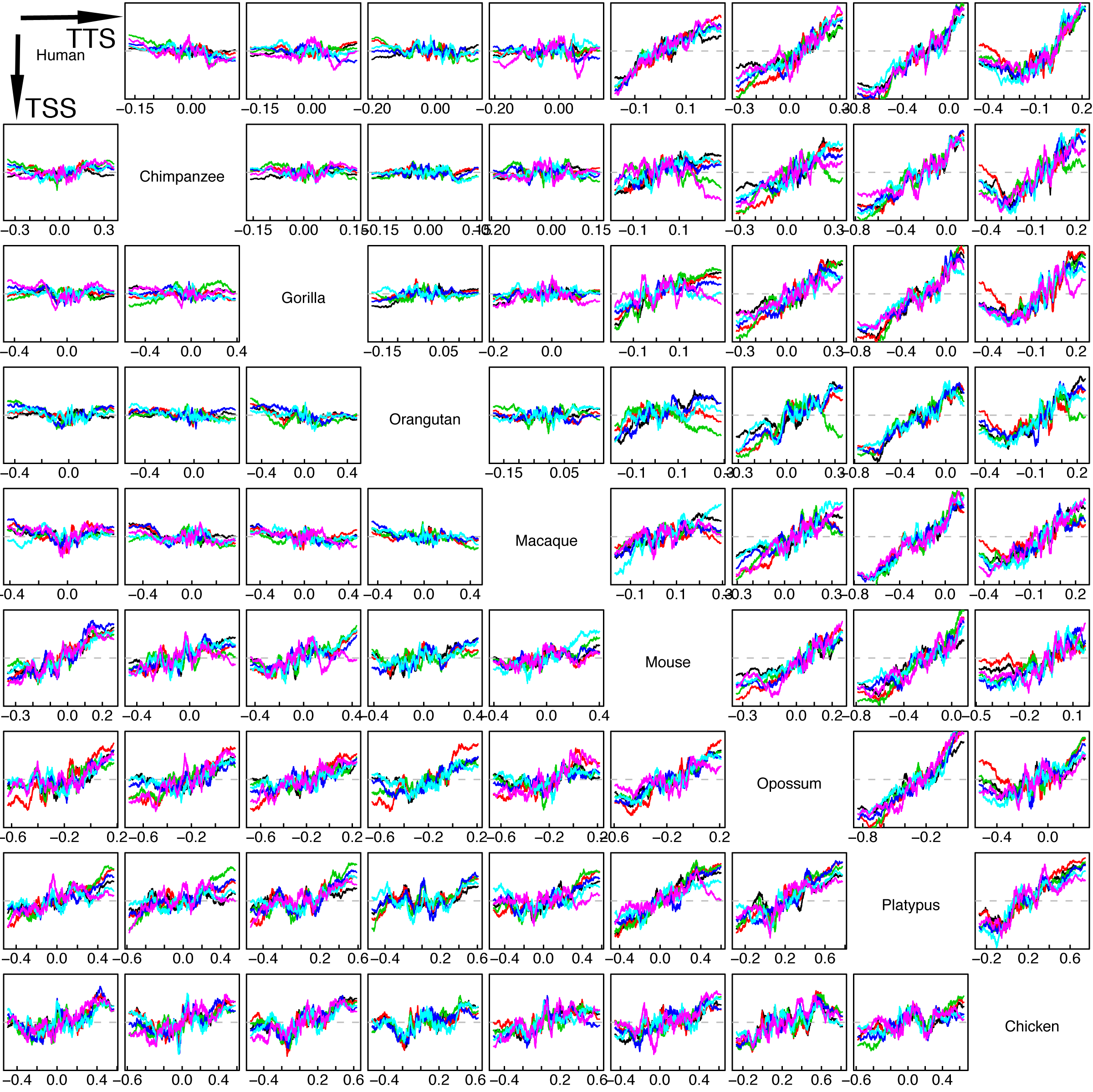

Supplement: Supplementary file 4 — Additional file 4: Expression levels of HCP and LCP genes in different tissues of different organisms. (TIFF 948 KB) [file 12864_2013_6381_MOESM4_ESM.tiff]

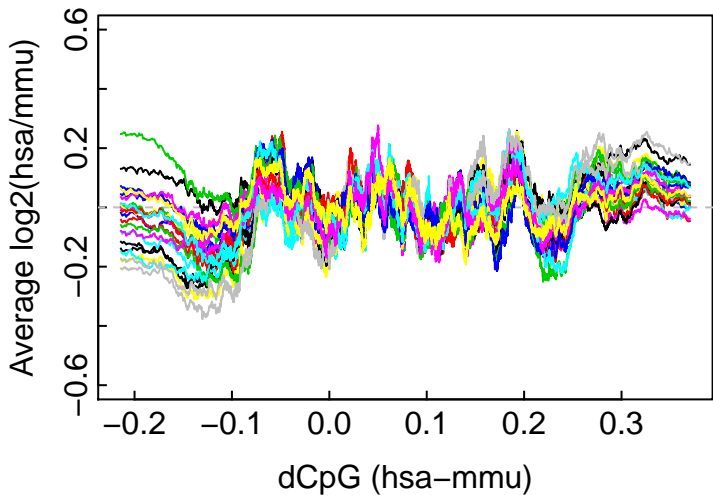

Supplement: Supplementary file 5 — Additional file 5: Correlation of normalized CpG content with microarray gene expression levels in 79 human tissues. (PDF 3 MB) [file 12864_2013_6381_MOESM5_ESM.pdf]
